# Public health interventions successfully mitigated multiple incursions of SARS-CoV-2 Delta variant in the Australian Capital Territory

**DOI:** 10.1017/S0950268823000201

**Published:** 2023-02-14

**Authors:** Robyn N Hall, Ashley Jones, Emma Crean, Victoria Marriott, Nevada Pingault, Alexandra Marmor, Timothy Sloan-Gardner, Karina Kennedy, Kerryn Coleman, Vanessa Johnston, Benjamin Schwessinger

**Affiliations:** 1 Research School of Biology, The Australian National University, Acton, Canberra, Australian Capital Territory, Australia; 2 COVID-19 Response Division, AC1850T Health Directorate, Canberra, Australian Capital Territory, Australia; 3 CSIRO Health & Biosecurity, Acton, Canberra, Australian Capital Territory, Australia; 4 Department of Clinical Microbiology and Infectious Diseases, Canberra Health Services, Australian National University Medical School, Canberra, Australian Capital Territory, Australia

**Keywords:** Australian Capital Territory, COVID-19, genomic epidemiology, pandemic, SARS-CoV-2

## Abstract

The COVID-19 pandemic has presented a unique opportunity to understand how real-time pathogen genomics can be used for large-scale outbreak investigations. On 12 August 2021, the Australian Capital Territory (ACT) detected an incursion of the SARS-CoV-2 Delta (B.1.617.2) variant. Prior to this date, SARS-CoV-2 had been eliminated locally since 7 July 2020. Several public health interventions were rapidly implemented in response to the incursion, including a territory-wide lockdown and comprehensive contact tracing. The ACT has not previously used pathogen genomics at a population level in an outbreak response; therefore, this incursion also presented an opportunity to investigate the utility of genomic sequencing to support contact tracing efforts in the ACT. Sequencing of >75% of the 1793 laboratory-confirmed cases during the 3 months following the initial notification identified at least 13 independent incursions with onwards spread in the community. Stratification of cases by genomic cluster revealed that distinct cohorts were affected by the different incursions. Two incursions resulted in most of the community transmission during the study period, with persistent transmission in vulnerable sections of the community. Ultimately, both major incursions were successfully mitigated through public health interventions, including COVID-19 vaccines. The high rates of SARS-CoV-2 sequencing in the ACT and the relatively small population size facilitated detailed investigations of the patterns of virus transmission, revealing insights beyond those gathered from traditional contact tracing alone. Genomic sequencing was critical to disentangling complex transmission chains to target interventions appropriately.

## Introduction

After the emergence of SARS-CoV-2 in late 2019 in Asia, the Australian Government declared a human biosecurity emergency on 18 March 2020 and closed its international borders to non-permanent residents and non-citizens on 20 March 2020 [1]. This largely restricted the impacts of the COVID-19 pandemic during 2020 and early 2021, compared to much of the rest of the world. Australia experienced a first wave of local SARS-CoV-2 transmission, with the so-called ‘ancestral’ SARS-CoV-2 variant, from March to April of 2020, predominantly driven by returning overseas travellers and cruise ship passengers [2]. A second wave, again due to ‘ancestral’ SARS-CoV-2, was experienced in June to October of 2020, primarily in the south-eastern state of Victoria (VIC) [3]. During the first and second waves, the Australian Capital Territory (ACT) experienced relatively little community transmission of SARS-CoV-2 (29 cases to 3 January 2021) [4]; the last local transmission prior to the Delta outbreak occurred on 7 July 2020 [5].

The ACT is a small (2358 km^2^) enclave within New South Wales (NSW) in south-eastern Australia with a population of approximately 453 558 people. During the first half of 2021, there were no local COVID-19 restrictions (i.e. density limits, mask wearing) and an elimination strategy (‘trace, test, isolate and quarantine’) was in place pending the rollout of vaccines to the eligible population. Pathogen genomics was of interest, but had not previously been used for large-scale outbreak investigation in the ACT and it was not known how this may complement traditional epidemiological contact tracing.

**Fig. 1.**
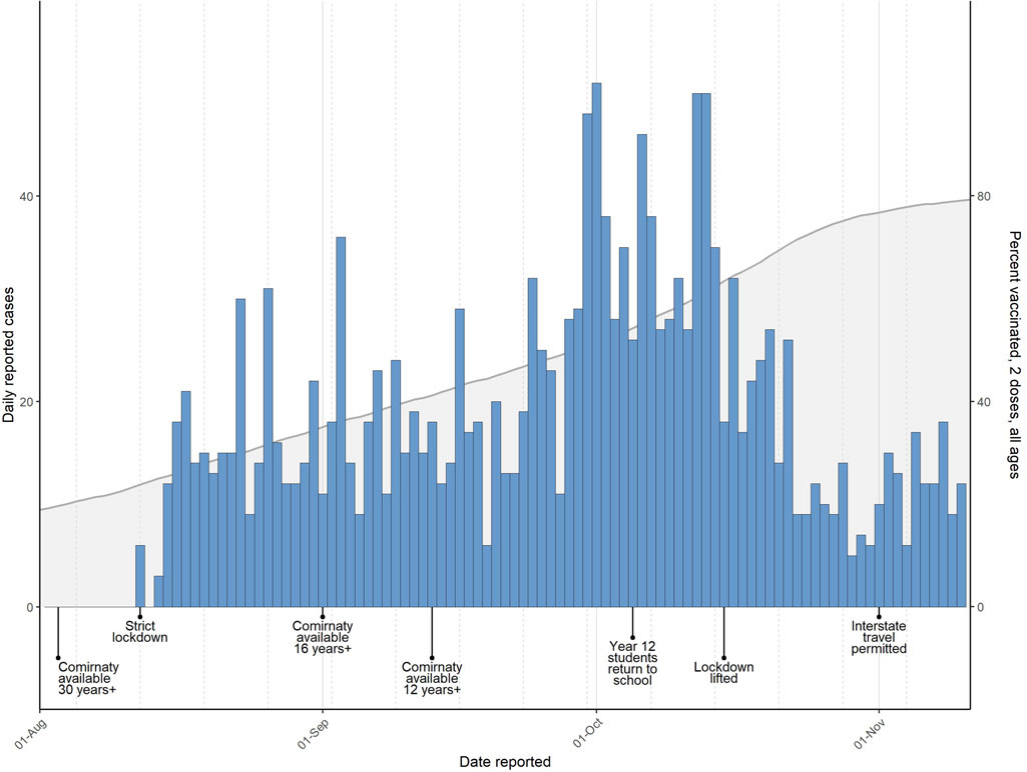
Epidemic curve and timeline of public health interventions for SARS-CoV-2 in the Australian Capital Territory, August to November 2021. The number of reported cases (left *y*-axis) per day is shown as a bar chart. The cumulative percentage of the total population receiving two vaccine doses (right *y*-axis) is shown as a grey area curve. A timeline of the major public health interventions is shown below the charts.

On 12 August 2021, a case of SARS-CoV-2 was detected in the ACT, 398 days after the last local transmission. This triggered a local lockdown, strict enforcement of mask mandates and the use of a QR code check-in application (Fig. 1). All cases underwent contact tracing, and cases and close contacts (defined as (i) a member of the same household, or (ii) a person notified by an authorised person that they were a close contact) were required to isolate or quarantine, respectively, for 14 days. Despite these interventions, the ACT subsequently experienced its first large-scale outbreak of COVID-19.

To investigate the utility of pathogen genomics for outbreak investigation in the ACT, full genome sequencing of SARS-CoV-2 was attempted for most cases. The study ran for 3 months, by which time most restrictions had eased. Lockdown was lifted on 15 October, due to high vaccination coverage and decreasing case counts (Fig. 1) [6]. On-campus learning for schools returned in a staged approach between 18 October and 1 November. Remaining restrictions such as density limits and mask requirements (in most settings) were lifted on 12 November; at this time, 95% of ACT residents aged 12 and over (81% of the total population) had received two COVID-19 vaccinations.

This was the first application of real-time genomic epidemiology at the local level in the ACT. We aimed to understand whether pathogen genomics could be implemented locally during a large-scale infectious disease outbreak and to determine how genomic epidemiology could be used to support traditional contact tracing and other public health interventions in the ACT context.

## Methods

### Sampling

For this study, cases were restricted to the 3-month period between 12 August 2021 and 11 November 2021, when public health interventions were in place [7]. All laboratory-confirmed SARS-CoV-2 cases in the ACT were notified to ACT Health, and core and enhanced epidemiological, demographic and clinical data were collected through telephone interviews using a standard questionnaire. Ethnicity information for positive cases was self-reported based on the question ‘How would you describe your ethnic or cultural background?’ using the Australian Bureau of Statistics Australian Standard Classification of Cultural and Ethnic Groups [8]. Vaccination data were obtained from ACT Health and population denominators were obtained from the Australian Bureau of Statistics. Positive RNA extracts from the two major local testing laboratories, ACT Pathology and Capital Pathology, were forwarded for pathogen sequencing at the Australian National University. We were not able to obtain specimens for some cases that were tested in other jurisdictions. Conversely, we received 30 specimens that were not classified as ACT cases but were included in our analyses. From 29 September we did not attempt sequencing of samples with RT-qPCR cycle thresholds ≥33 or from cases that were household contacts of a sequenced case.

### SARS-CoV-2 sequencing

SARS-CoV-2 sequencing was performed using the ARTIC Network amplicon sequencing protocol, using either Superscript IV (Life Technologies) or Lunascript (New England Biolabs) reverse transcriptase for cDNA synthesis [9]. For PCR amplification, we initially used the v3 primer set until 10 October 2021, when we switched to v4 [10]. Pooled amplicon libraries were prepared using the Ligation Sequencing Kit (Oxford Nanopore Technologies (ONT, United Kingdom) EXP-AMII001) and native barcodes (ONT EXP-NBD196). Sequencing was performed on a MinION Mk1B using a R9.4.1 FLO-MIN106D flow cell, as per manufacturer's instructions (ONT). Sequencing progress was monitored with RAMPART [11] until sufficient coverage was achieved. Raw fast5 sequencing reads were basecalled to fastq and demultiplexed using Guppy (versions 4 and 5, ONT). Consensus sequences were generated using the ARTIC network SARS-CoV-2 bioinformatics pipeline version 1.2.1 against the MN908947.3 reference sequence [12]. EPI_ISL_3643665 was processed and sequenced using Illumina technology at the Microbial Genomics Reference Laboratory, NSW Health Pathology (NSWHP) Institute of Clinical Pathology and Medical Research. EPI_ISL_5587718 was processed and sequenced using the Oxford Nanopore MinION at NSWHP Royal Prince Alfred Hospital.

Sequencing turnaround time was estimated by calculating the time from sample collection to preliminary reporting of the sequencing results to ACT Health. Since no time stamp was available for sample collection, we used 12:00.

### Phylogenetic analysis

Sequences with ≤20% ambiguous bases were aligned against the Wuhan-1 (GenBank Accession NC_045512 [13]) reference sequence using the FFT-NS-2 algorithm in MAFFTv7.450 [14] as implemented in Geneious Prime 2021.2.2 (https://www.geneious.com/). The alignment was curated manually to check for gaps and misalignments. Sequences were assigned to ACT genomic lineages and sublineages based on neighbour-joining phylogenies (≤10% ambiguous bases only) constructed using the Tamura-Nei model as implemented in the Geneious Tree Builder in Geneious Prime 2021.2.2 and/or on manual exploration of the alignments at lineage-defining sites. Lineage assignment was revised daily during the COVID-19 response, based on the incorporation of new sequences and in conjunction with epidemiological information. Comparisons to publicly available Australian and international sequences were performed with the UShER webserver [15].

To estimate a time-scaled phylogeny, a maximum likelihood (ML) phylogeny was estimated using iqtreev2.1.2 [16] for sequences with ≤10% ambiguous bases (*n* = 1275). Branch support was estimated using 1000 ultrafast bootstrap approximations [17] and 1000 replicates of the SH-like approximate likelihood ratio test [18]. This ML tree, along with sample collection dates, was used as input for treetimev0.8.5 [19]. The tree was rooted on NC_045512 (https://www.ncbi.nlm.nih.gov/genbank/).

Figures were generated in Rv4.1.0 using the following packages: tidyversev1.3.1 [20], ggtreev3.3.0.900 [21], scalesv1.1.1 [22], ggaltv0.4.0 [23] and cowplotv1.1.1 [24].

## Results

### Rapid implementation of pathogen genomics during a large-scale infectious disease outbreak

From 12 August 2021 to 11 November 2021, 1793 laboratory-confirmed SARS-CoV-2 infections were reported in ACT residents. We were able to attempt SARS-CoV-2 sequencing for 1438 cases (80%). Near-complete genomes (≤1% ambiguous bases) were recovered from 287 cases (16.0% of all ACT cases), we recovered 960 partial genomes (1% to ≤10% ambiguous bases; 53.5% of all ACT cases), and 100 poor quality genomes (10% to ≤20% ambiguous bases; 5.6% of all ACT cases). The remaining 91 cases for which sequencing was attempted yielded incomplete genomes (>20% ambiguous bases). The estimated turnaround time for sequences and analyses to become available for public health action was within 3.2 days of sample collection for 50% of sequences, and within 7.0 days of sample collection for 95% of sequences. Additionally, we sequenced 30 samples from non-ACT cases that were received through ACT laboratories.

### Genomic epidemiology revealed multiple incursions into the ACT with distinct demographics

**Fig. 2.**
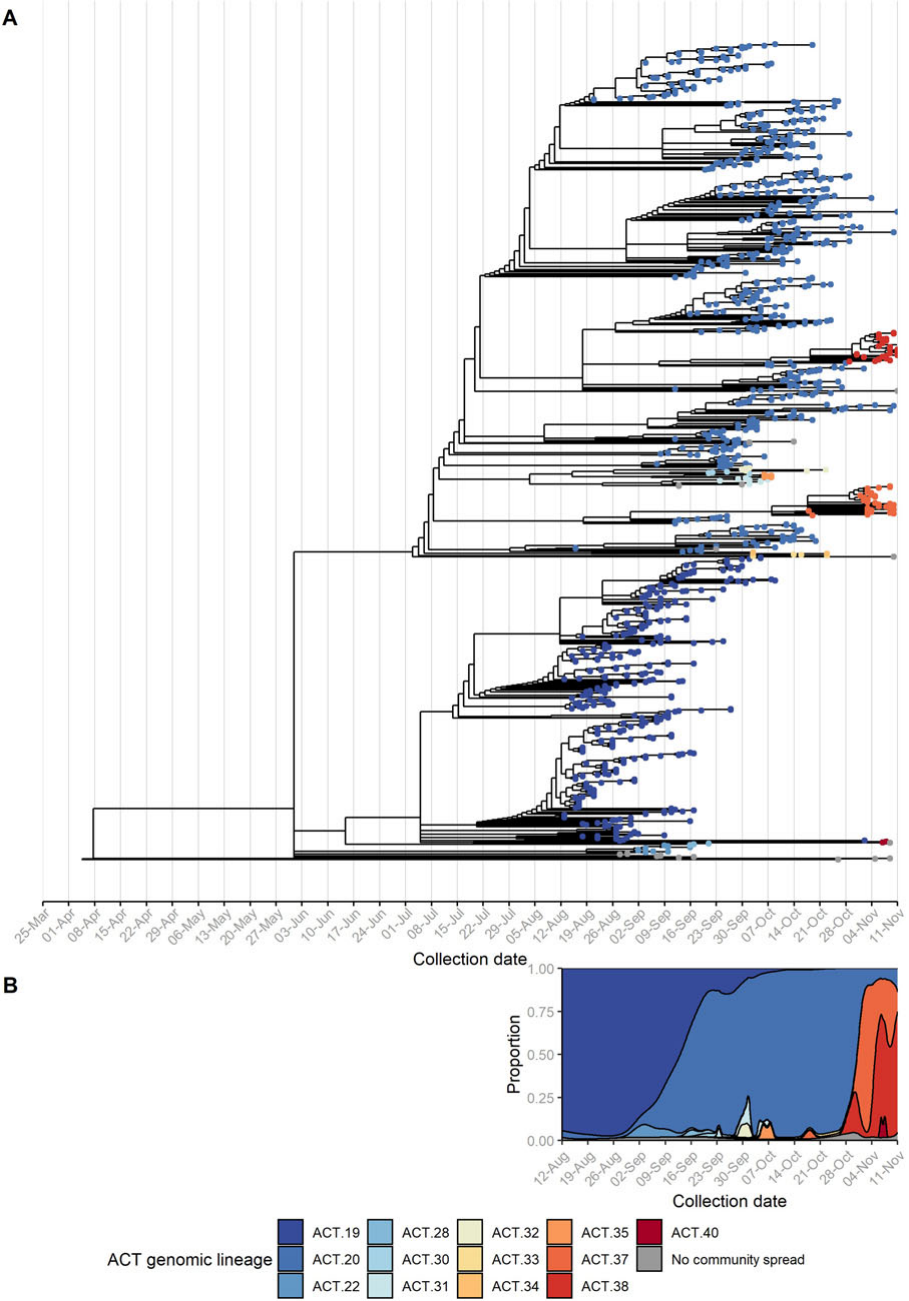
Incursions and onward spread of SARS-CoV-2 B.1.617.2 (Delta) in the Australian Capital Territory (ACT), 12 August to 11 November 2021. Sequencing of SARS-CoV-2 was attempted on 80% of ACT cases reported during the study period and an additional 30 non-ACT cases. A time-structured phylogeny was estimated based on consensus sequences with ≤10% ambiguous bases (a). Tips are coloured by ACT genomic lineage. Each lineage reflects a separate incursion event with subsequent local spread, as defined by phylogenetic analysis and corroborating epidemiological information. Sequences where onwards transmission within the community was not identified within the ACT are coloured grey. The density plot shows the relative proportion of ACT genomic lineages over time, based on all sequences with ≤20% ambiguous bases (b). Both (a) and (b) are scaled to the same *x*-axis.

Based on the phylogeny and corroborating epidemiological data, we identified at least 13 incursions into the ACT resulting in forward transmission in the community over the study period, despite strict interstate travel restrictions. Each incursion was classified as a separate ACT genomic lineage (Fig. 2). Furthermore, we identified 13 sequences as genomic singletons that did not cluster (≤2 nucleotide differences) with another ACT sequence, and three lineages that were contained to a single household. These introductions did not result in forward transmission within the community.

The individual incursions led to ongoing transmission chains that varied considerably in size, duration and demographics (Table 1). The Delta (B.1.617.2) ‘wave’ in the ACT was dominated by two large incursions, ACT.19 and ACT.20, both initially identified early in the outbreak (12 and 19 August, respectively). To assist with active case finding and control efforts, a mobile (in-reach) testing and vaccination strategy was rolled out by partnering with health services and non-government organisations. By mid-September, the ACT.19 incursion had been mostly controlled, at which point ACT.20 was detected more widely. ACT.20 case numbers declined throughout October, which negatively correlated with the number of vaccine doses delivered (Fig. 1) [6]. However, sporadic cases continued to be identified through to the end of the study period.

**Table 1.** Size, duration and epidemiological characteristics of SARS-CoV-2 B.1.617.2 (Delta) incursions

ACT genomic lineage	Number of cases	First detection	Duration (days)	Median age (years)	Per cent not identified as Australian/English^a^
ACT.19	478	12 August	65	24.4	80
ACT.20	724	15 August	88^b^	32.3	45
ACT.22	13	01 September	14	23.0	75
ACT.28	3	15 September	5	21.1	100
ACT.30	4	20 September	11	44.3	100
ACT.31	13	23 September	13	33.8	50
ACT.32	7	29 September	2	22.3	33
ACT.33	5	02 October	20	37.5	25
ACT.34	3	02 October	20	31.1	33
ACT.35	7	05 October	2	16.3	50
ACT.37	47	17 October	25^b^	7.9	17
ACT.38	46	28 October	14^b^	19.2	50
ACT.40	2	07 November	1^b^	33.8	50

a Ethnicity was classified using self-reported ethnic and cultural identification data into two groups; those who identified as Australian/English, and those who identified with another cultural and/or ethnic group/s. Ethnicity data were not available for 26 sequenced cases (2.0%).

b Denotes ACT genomic lineages still active at the end of the study period (i.e. detected within 2 weeks of the end of the study period).

Cases related to the ACT.19 incursion frequently were from large ethnically diverse households with non-nuclear families, often spanning multiple residences (Table 1). For comparison, while the Australian Bureau of Statistics does not report ethnicity data directly, in the 2021 national census 24.6% of ACT persons in the ACT reported speaking a language other than English at home and 29% reported being born overseas. The number of people per household was relatively high and communication and community engagement was complex (Supplementary Fig. 1). Additionally, many were essential workers, often with carer responsibilities, resulting in forward transmission into other vulnerable populations. To assist with control efforts, ACT Health engaged extensively with community and cultural leaders, cross-government and non-government agencies, and focussed efforts on the provision of culturally appropriate and in-reach supports.

In contrast, the ACT.20 incursion predominantly affected a less ethnically diverse and more socially disadvantaged cohort (Table 1). While there were often fewer people per household in this group (Supplementary Fig. 1), there were challenges around contact tracing and access to testing.

The phase after the lifting of the territory-wide lockdown (on October 15) was characterised by two medium-sized incursions, ACT.37 and ACT.38. The median age of cases in these incursions was 8 and 19 years, respectively (Table 1); this was significantly lower than the median age of non-ACT.37 or ACT.38 cases (i.e. all cases belonging to other ACT genomic lineages or genomic singletons), which was 28 years (Mood's test, adjusted *P* = 5 × 10^−3^ and 2 × 10^−5^, respectively). Contact tracing revealed that one of these incursions was linked to a school setting [25], while the other was attributed to a party at a private residence [26]. Genomic sequencing showed that the school-associated incursion was limited to students and their immediate contacts (e.g. parents, siblings and other household members); there was no extended community transmission related to this incursion. In contrast, the spread from the private party was more extensive.

### Genomic epidemiology supported contact tracing by resolving complex transmission chains and identifying potential exposure sources

**Fig. 3.**
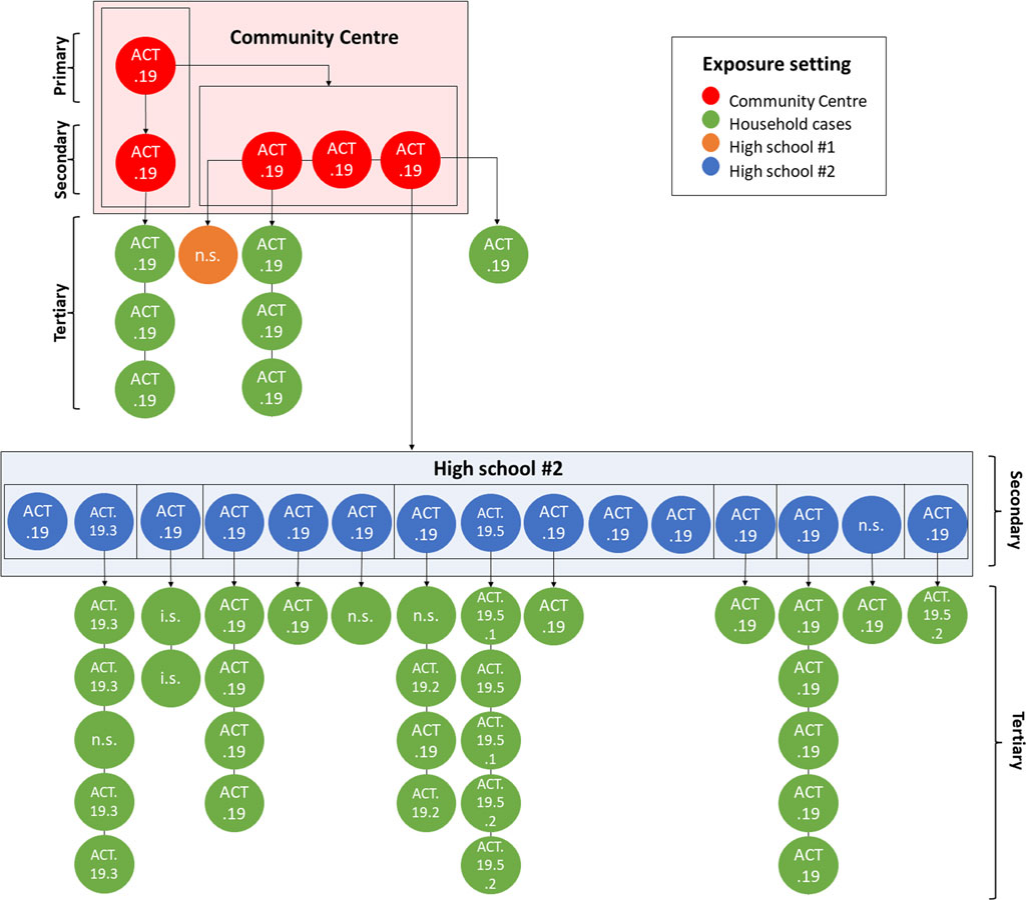
Resolution of a complex transmission chain using genomic epidemiology. Dots represent individual cases and are coloured by exposure setting. Primary, secondary and tertiary cases are marked by braces. The ACT genomic sublineage of each case is specified. Directionality of transmission, inferred from epidemiological contact tracing, laboratory information and/or genomic sequencing, is indicated by arrows. Boxes delineate separate cohorts. n.s., Not sequenced; i.s., case diagnosed interstate.

In addition to the tracking of broad-scale ACT genomic lineages, we used single mutations to define genomic sublineages. These were found to map closely to epidemiologically defined case clusters and this sublineage information was used to link cases with an unknown source of acquisition to clusters and to resolve complex transmission chains, such as where cases were linked to multiple exposure locations. For example, genomic sequencing revealed links between two different high schools via common exposure through extra-curricular activities (Fig. 3) [27].

**Fig. 4.**
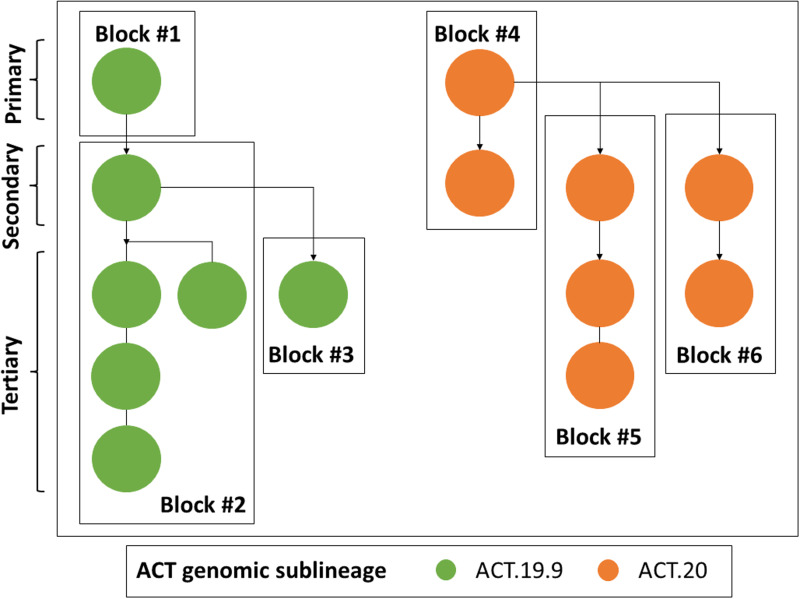
Genomic sequencing revealed two independent incursions into a social housing complex over a 3-week period. Dots represent individual cases and are coloured by ACT genomic sublineage. Primary, secondary and tertiary cases are marked by braces. Directionality of transmission, inferred from epidemiological contact tracing, laboratory information and/or genomic sequencing, is indicated by arrows. Boxes delineate separate cohorts.

Incorporating this genomic contact tracing, we were able to identify transmission at certain exposure sites and implement enhanced infection control measures in these settings. For example, genomic information showed that a case cluster at a social housing complex was the result of several incursions over a 3-week period, rather than a single superspreading event which was assumed based only on case interviews and contact tracing information (Fig. 4). While isolated cases were identified in other high-risk settings, such as hospitals and correctional facilities, genomic sequencing showed that typically these cases were community-acquired and that in most of these settings (apart from a single outbreak in a residential aged care facility) there was no significant spread.

## Discussion

A key feature of the Delta (B.1.617.2) SARS-CoV-2 outbreak in the ACT was the very high proportion of cases for which sequencing was attempted (>80% of all reported cases) with exceptional short turnaround time from sample collection, leading to a detailed and timely understanding of local transmission networks and number of distinct incursions. The ACT is one of the few jurisdictions globally that was able to maintain such high rates of SARS-CoV-2 sequencing for an extended period [28–30]. The rapid turnaround time for sequencing results (<7.0 days in 95% of cases and <3.2 days in 50% of cases) greatly facilitated epidemiological contact tracing and the identification of high-risk exposure locations.

Through our genomic analyses, we were able to determine that the ACT Delta ‘wave’ was driven by two separate dominant incursions, ACT.19 and ACT.20. This insight allowed us to explore the demographic factors contributing to the ‘success’ of these incursions compared to incursions that did not transmit extensively. Both dominant incursions particularly impacted vulnerable groups within the community, where crowded living arrangements and lower health literacy were contributing factors; however, the specific demographic characteristics of these vulnerable populations were distinct. The two major incursions varied considerably in the demographic groups affected, and subsequently in duration and total number of cases. Several studies have already demonstrated a disproportionate risk of SARS-CoV-2 infection in socioeconomically disadvantaged groups and in ethnic minorities [31–35], and it is well-established that socioeconomic factors are generally important determinants of health and disease. The use of genomic epidemiology to differentiate between the two overlapping incursions led to a greater understanding of transmission in at-risk populations and the need for enhanced mitigation measures in these settings, such as culturally appropriate engagement and the deployment of in-reach interventions in association with non-government organisations.

Interestingly, the first cases of ACT.20 were detected on 15 August 2021, 3 days after the detection of ACT.19. Yet cases only began to increase exponentially in early September and there was very limited genetic diversity in the month following detection, with the first mutation detected in samples collected on 11 September 2021. It is possible that there was undetected transmission of ACT.20 after the first detection, although testing rates were high, there was exhaustive contact tracing of cases, and strict lockdowns were in place. Alternatively, there may have been a separate introduction of a genetically identical virus. Our findings constrain the lower bound of the number of possible incursions. The genetic diversity of SARS-CoV-2 circulating in Australia during mid-2021 was limited, since all local cases arose from a single point source outbreak in Sydney, NSW, on 15 June 2021 [36]. Indeed, of the publicly available Australian sequences, over 400 of these are identical to the ACT.20 sequence, and it is very likely that many more cases were either not detected, not successfully sequenced or not shared publicly. Therefore, multiple incursions of near-identical SARS-CoV-2 genomes would likely have been missed. This highlights the challenges around identifying individual incursions early in an outbreak when genetic diversity is limited [37–39]. The many genomic singletons identified that did not result in forward transmission within the community were likely contained through the strict 14-day quarantine restrictions for returning residents.

Notably, the two dominant incursions during the resolution of ACT.20 (i.e. ACT.37 and ACT.38) disproportionately affected incompletely vaccinated younger persons, with transmission occurring at (i) a large private party, and (ii) within a school setting, demonstrating the marked impacts of even superspreading events. Of relevance, schools reopened between 5 October and 1 November and vaccines were not available for those aged 16–18 years until 1 September, and for those aged 12–16 years until 13 September. The cohorting strategy applied in the school setting as students and teachers returned to on-campus learning, in addition to other COVID-safe measures such as masking, was very effective in limiting the extent of community transmission associated with this incursion.

While we observed complete replacement of ACT.19 by ACT.20, and near-complete replacement of ACT.20 by ACT.37 and ACT.38, these replacements were not a consequence of enhanced epidemiological fitness of any of these viruses. Indeed, the ACT.20 founder sequence had only five non-synonymous changes relative to the ACT.19 founder sequence across all coding sequences, only one of which was in the spike protein, while ACT.37 and ACT.38 each had two non-synonymous changes relative to ACT.20, none of which were in the spike protein. The spread of SARS-CoV-2 Delta in the ACT is a clear example of repeated founder effects, because of the extensive mitigation measures and the stochastic nature of transmission of SARS-CoV-2 (overdispersion) [40]. This could only be revealed through high levels of genomic sequencing in this relatively small population.

By 11 November (the end of the study period) all incursions were considered to be sufficiently controlled and population vaccination coverage was high, leading to the lifting of most restrictions. Notably, during the study period, there were 11 COVID-19-related deaths. The extensive contact tracing and case follow-up employed in the ACT likely facilitated the early identification of those cases eligible for enhanced treatment, such as monoclonal antibody therapies, which became available in Australia from late August. The high vaccination rates achieved in the ACT and good compliance with public health social measures during the outbreak period likely contributed to the low observed mortality [6].

Our study revealed that pathogen genomics could be rapidly implemented during a large-scale infectious disease outbreak and highlighted the utility of intensive genomic sequencing to support traditional contact tracing and other public health interventions in real time. We determined that the ACT Delta outbreak was driven by several independent incursions, with successive waves impacting different vulnerable groups. However, by deploying enhanced interventions (such as culturally appropriate engagement and partnered in-reach interventions) into these at-risk communities, timely public health measures were successful in mitigating these incursions and most importantly, in preventing severe clinical outcomes. The combination of stringent and timely public health interventions, exhaustive contact tracing (due to the relatively small population size) and high levels of viral sequencing close to real time effectively limited the two major Delta incursions, ACT.19 and ACT.20, within a 3-month period.

## Data Availability

All sequences are available in GISAID. Accession IDs are provided in Supplementary Table 1.

## Supplementary material

The supplementary material for this article can be found at https://doi.org/10.1017/S0950268823000201.
